# Cone Beam CT Assessment of Mandibular Foramen and Mental Foramen Positions as Essential Anatomical Landmarks: A Retrospective Study in Vietnam

**DOI:** 10.7759/cureus.59337

**Published:** 2024-04-30

**Authors:** Lam N Le, Thao T Do, Loc T Truong, Anh T Dang The, My H Truong, Duyen K Huynh Ngoc, Luan M Nguyen

**Affiliations:** 1 Department of Pediatrics Dentistry and Orthodontics, Faculty of Odonto-Stomatology, Can Tho University of Medicine and Pharmacy, Can Tho, VNM; 2 Department of Oral Pathology and Microbiology, Can Tho University of Medicine and Pharmacy, Can Tho, VNM; 3 Faculty of Odonto-Stomatology, Can Tho University of Medicine and Pharmacy, Can Tho, VNM

**Keywords:** radiology research, inferior alveolar nerve, cone beam computed tomography, mental foramen, mandibular foramen

## Abstract

Introduction: The mandibular foramen (MnF) and the mental foramen (MF) are essential anatomical landmarks that should be considered before any surgical procedures in the mandible. This study aimed to investigate the characteristics of the MnF and MF in relation to adjacent anatomical structures, as well as age and gender differences, using cone beam computed tomography (CBCT) projections.

Methods: The study was conducted from August 2023 to January 2024 at the Can Tho University of Medicine and Pharmacy Hospital, Vietnam. In this retrospective study, 50 CBCT images of Vietnamese patients were randomly taken for various clinical purposes. Furthermore, relevant data, such as gender and age groups, were selected to evaluate the correlations, along with specific inclusion criteria. Patients within the age range of 18-69 with a symmetrical mandible were included.

Results: The distance of the MnF-MN was 29.6±5.0 mm (right) and 30.1±4.6 mm (left) in males and 25.0±4.2 mm (right) and 26.3±5.0 mm (left) in females. The distance of the MnF-posterior border of the ramus (P) was 16.2±3.6 mm (right) and 15.0±2.3 mm (left) in males. For females, it was 17.1±2.9 mm (right) and 13.8±1.7 mm (left). The distance of the MF-body mandible (MB) was 15.4±2.4 mm (right) and 15.6±2.0 mm (left) in males and 14.0±2.1 mm (right) and 14.3±1.6 mm (left) in females. The distance of the MF-mandibular midline (MM) was 27.0±2.6 mm (right) and 27.0±2.9 mm (left) in males and 25.3±2.0 mm (right) and 25.1±2.2 mm (left) in females. These distances showed statistically significant differences depending on gender (P<0.05).

Conclusion: It can be said that CBCT provides comprehensive information about the MnF and the MF for dentists in research and clinical practice.

## Introduction

Anatomically, the mandibular foramen (MnF) is an irregular opening that is slightly posterior on the internal surface of the ramus [1,2]. It is located in the least mobile area during the normal process of opening and closing the mouth, so it has the effect of protecting nerves and blood vessels. Additionally, the mental foramen (MF) is located bilaterally at the outer surface of the mandibular in the area below the premolars [3,4]. MF is usually located midway between the base of the mandible and the alveolar bone crest in the same vertical plane of the infraorbital foramen [5]. These foramina are important openings for the vascular bundle of the inferior alveolar neurovascular bundle to enter and exit [6].

Although uncommon, these foramina have anatomical variations not only in their size and shape but also their the location and direction of the opening. The variations in MnF and MF have been reported, and if unnoticed, injury may lead to patient morbidity, neurosensory disturbances, and other undesired complications [4,7]. As such, a proper understanding of vital anatomical structures and proper planning are the keys to achieving successful procedures [7,8].

The MnF and the MF are essential anatomical landmarks that should be considered before any surgical procedures in the posterior mandible, such as inferior alveolar nerve (IAN) block anesthesia procedures, preventing the complications to surgery involving mandibular molars, mandibular implant treatment, mandibular osteotomies, and orthognathic surgery [9,10]. Avoiding damage to the neurovascular bundle while obtaining appropriate access to accomplish the procedure with excellence requires a keen awareness of anatomic structures, including both the MnF and MF [7].

The recent introduction of cone beam computed tomography (CBCT) enables a three-dimensional evaluation by the reconstruction of high-resolution images and the acquisition of images through a one-time rotation at a lower cost, a lower dose of radiation, and with easier operation, as compared with conventional multislice CT [11]. It is critically important to accurately identify the position of the MnF and MF in the clinical setting. It has also emphasized that the knowledge of the location of the MnF would assist doctors or surgeons in the oral disease diagnosing and treatment process [9,10,12,13]. Over the last decade, CBCT has become a standard for imaging for dental implant assessment, endodontic evaluation, and various jaw lesions [14]. However, measurements related to the MnF on CBCT have not been performed clinically in common in Vietnam until now because they require highly specialized techniques. Additionally, radiographically, the MF normally appears as a single structure. It is observed as a rounded or oval radiolucent area at the level of the lower premolar apices or superimposed on them [15].

This study aimed to investigate the characteristics of MnF and MF in relation to adjacent anatomical structures, as well as age and sex differences in CBCT projections.

The position of MnF and MF can vary in each person, especially with age; to avoid the possible influence of this change in location, in this study, only patients between 18 and 69 years of age were included [10].

## Materials and methods

Study participants

The inclusion criteria included completely voluntary patients, in the age range of 18-69, and who have a symmetrical mandible; all of the CBCT images required high qualification to identify detailed structures in the mandible with a CBCT voxel size of approximately 0.3 mm. Furthermore, the patients with insufficient information or pathological mandibular lesions, a history of mandibular fractures, surgery, and/or artifacts due to metal parts were excluded [5,9,16-19].

Study methods

The study was approved by the Research Ethics Committee, Can Tho University of Medicine and Pharmacy, Vietnam, to select the sample using the convenience sampling method. In this retrospective study, 50 CBCT images of Vietnamese patients were randomly taken for various clinical purposes in 2023 and earlier. The study was conducted from August 2023 to January 2024 at Can Tho University of Medicine and Pharmacy Hospital. The prevailing data, such as gender and age groups (18-25 years old, 26-40 years old, 41-55 years old, and 56-69 years old), were also chosen to evaluate the correlation.

Study procedures and indicators

**Figure 1:**
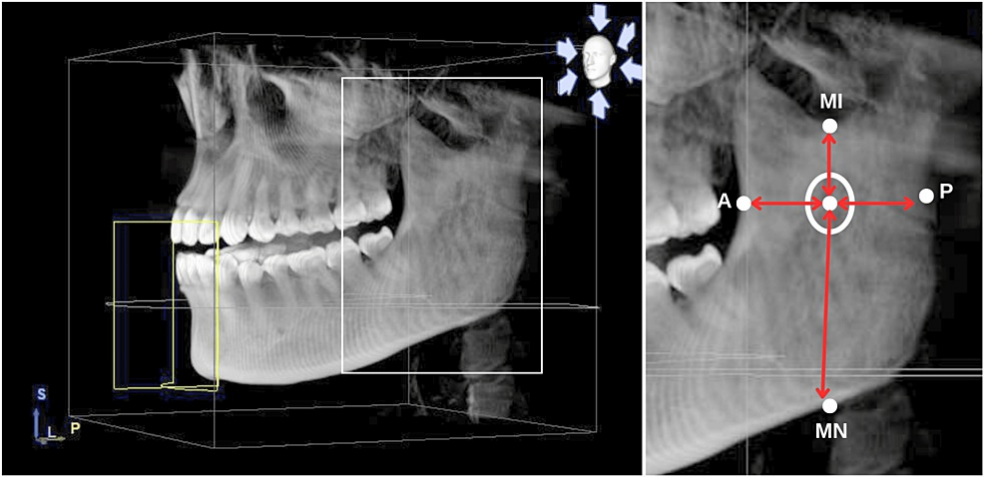
Comparing the distance between the mandibular foramen and anatomical landmarks. A - anterior border of the ramus, P - posterior border of the ramus, MI - mandibular incisura, MN - mandibular notch

**Figure 2:**
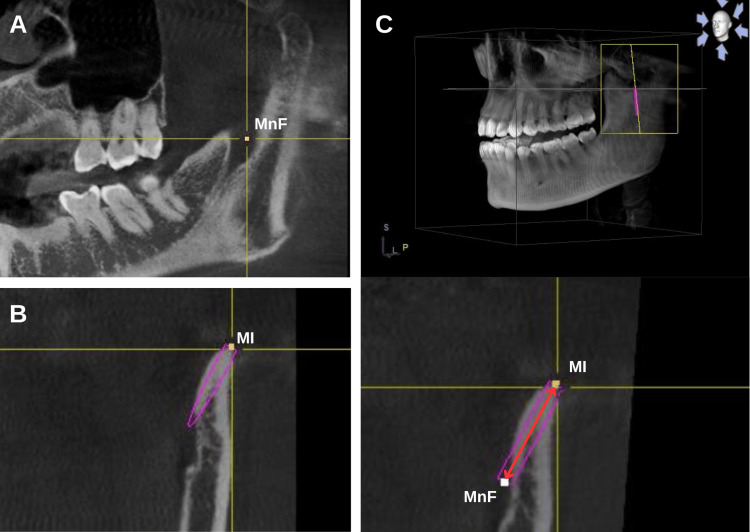
Measurement method. A. Cross-sectional tomography with appropriate axial rotation containing the mandibular foramen (MnF). B. Plane containing the lowest point of the mandibular incisura (MI). C. Plane containing the two points to be measured for distance (MnF-MI)

To begin with, the distances from the center of MnF to the following sites were determined: distance from the center of the MnF to the deepest point of the anterior (MnF-A), distance from the center of the MnF to posterior borders of the ramus (MnF-P), distance from the center of the MnF to the most superior point of the curvature of the mandibular notch (MnF-MN), and distance from the center of the MnF to the inferior point of the mandibular incisura (MnF-MI) (Figure 1). The first stage was using a coronal plane and rotating the appropriate axial in order to find the MnF, marked number 1, and then adding a nerve point in the toolbar, which was used to highlight the mandibular canal. The following steps include measuring the remainder of the landmarks in the appropriate frontal plane, marked number 2. From the sagittal plane, moving the appropriate axis until displaying the two points marked above, select the dimension tool to calculate the distance (Figure 2). Lastly, the indicator measurements were processed by using Sidexis, GALILEOS software (version 1.8, Sirona, Germany). The data source was taken by a CBCT scanner: Sirona Orthophos SL 3D (Sirona, Germany).

**Figure 3:**
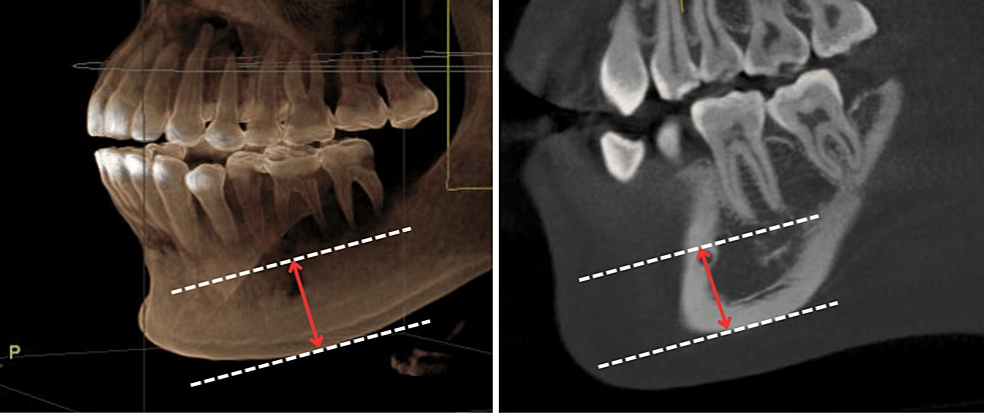
Distance from the superior border of the mental foramen to the inferior border of the body mandible (MF-MB).

**Figure 4:**
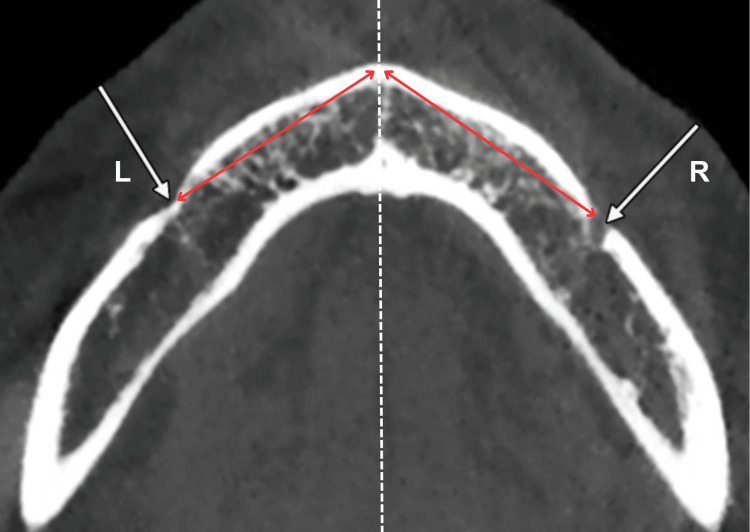
Distance from the anterior border of the mental foramen to the mandibular midline (MF-MM). L - Left, R - Right

The MF also obtained a markable target as the distance from the superior border of the MF to the inferior border of the body mandible (MB), which could be measured by the following instructions. Using a drawing tool named Measure Distance in GALILEOS software, draw a perpendicular line to two other parallel lines that were established at the superior border of the MF and the inferior border of the MB, respectively (Figure 3). The other target was the distance from the anterior border of the MF to the mandibular midline (MM) - characterized as a midline in the sagittal plane intersecting the tangential plane. The MM was selected based on the nasal spine because of the personal traits of maxillary and the situation of missing teeth. Therefore, the distance (MF-MM) was also understood as the distance from the anterior borders of the MF to the MM in the frontal plane. Then, apply the measurement methods the same as the MnF distances mentioned (Figure 4). The last step was done by the same techniques as measuring the indicators of MnF.

Statistical analysis

Assembling figures and data were imported into Microsoft Excel 2020 software (Microsoft® Corp., Redmond, WA). Then, SPSS Statistics 22.0 statistical software was used for the analysis. CBCT projections were taken by a dental radiologist technician and reevaluated by comparing them to different planes (frontal, transverse, sagittal) in a three-dimensional space. Some mistakes made during drawing and measuring processes on CBCT were inevitable unless all the above stages were verified by one examiner. This researcher's consistency was determined as follows: After the sampling was finished and after obtaining the total number of samples, there were 20 films randomly selected to be plotted and measured again with the same method by the same person two months later (test-retest method). The statistics collected from the second measurement were compared to those of the first time using the Pearson correlation coefficient.

## Results

All results were expressed as mean±standard deviation. This study consisted of 50 CBCT scans. The sample included 28 males and 22 females. The average age between males and females is 33.8±11. Considering the age group classification, the majority of age is 26-40.

Mandibular foramen 

The MnF-MI on the right was 18.3±3.1 mm, and that on the left was 17.5±3.3 mm in males. Meanwhile, it was 17.3±2.9 mm on the right and 17.0±2.5 mm on the left in females. The distance between the MnF and the MN was 29.6±5.0 mm on the right and 30.1±4.6 mm on the left in males. For females, it was 25.0±4.2 mm on the right and 26.3±5.0 mm on the left. This distance showed statistically significant differences according to gender and sides of the mandibular (P<0.05).

The distance of MnF-A on the right was 16.2±3.6 mm, and that on the left was 17.0±3.3 mm in males. Meanwhile, the distance of MnF-A was 16.2±3.6 mm on the right and 17.2±2.6 mm on the left in females. The distance of MnF-P was 16.2±3.6 mm on the right and 15.0±2.3 mm on the left in males. For females, it was 17.1±2.9 mm on the right and 13.8±1.7 mm on the left. This distance showed statistically significant differences according to gender and sides of the mandibular (P<0.05) (Table 1).

**Table 1:** The average distances from the center of MnF to various sites. Distance from the center of the mandibular foramen to the deepest point of the anterior (MnF-A), distance from the center of the mandibular foramen to posterior borders of the ramus (MnF-P), distance from the center of the mandibular foramen to the most superior point of the curvature of the mandibular notch (MnF-MN), and distance from the center of the mandibular foramen to the inferior point of the mandibular incisura (MnF-MI)

Distance	Gender	Distance (mm)	
		Right	Left
MF-MN	Male	18.3±3.1	17.5±3.3
	Female	17.3±2.9	17.0±2.5
	P value	0.244	0.604
MF-MI	Male	29.6±5.0	30.1±4.6
	Female	25.0±4.2	26.3±5.0
	P value	0.001	0.007
MF-A	Male	16.2±3.6	17.0±3.3
	Female	16.2±3.6	17.2±2.6
	P value	0.383	0.827
MF-P	Male	16.2±3.6	15.0±2.3
	Female	17.1±2.9	13.8±1.7
	P value	0.008	0.042
Independent samples test			

The results of the average distance of the MnF on both sides were calculated in relation to the groups of age, but no statistically significant difference was found (Table 2).

**Table 2:** The average distances between the MnF and anatomical landmarks according to age group classification. Distance from the center of the mandibular foramen to the deepest point of the anterior (MnF-A), distance from the center of the mandibular foramen to posterior borders of the ramus (MnF-P), distance from the center of the mandibular foramen to the most superior point of the curvature of the mandibular notch (MnF-MN), and distance from the center of the mandibular foramen to the inferior point of the mandibular incisura (MnF-MI)

	Age	MnF-MI (mm)	MnF-MN (mm)	MnF-A (mm)	MnF-P (mm)
Right	18-25	18.2±2.7	26.6±5.0	17.1±3.5	14.0±1.8
	26-40	18.0±3.3	26.5±5.0	15.7±2.8	15.1±2.1
	41-55	16.7±2.1	30.1±4.2	17.8±4.2	13.4±2.6
	56-69	18.2±4.6	32.3±5.8	17.6±2.2	15.3±1.7
	P value	0.151	0.516	0.341	0.219
Left	18-25	17.5±2.1	27.7±5.8	17.5±3.6	13.6±1.8
	26-40	17.8±3.3	27.9±4.5	16.5±2.3	14.8±2.0
	41-55	15.5±2.6	29.7±5.0	18.2±4.3	14.2±2.9
	56-69	17.1±3.3	31.8±6.4	17.0±0.9	15.9±1.5
	P value	0.588	0.310	0.636	0.143
ANOVA test					

MF

The distance from the superior border of the MF to the inferior border of the mandible (MF-MB) was 15.4±2.4 mm on the right and 15.6±2.0 mm on the left in males and 14.0±2.1 mm and 14.3±1.6 mm in females. The distance of MF-MM was 27.0±2.6 mm on the right and 27.0±2.9 mm on the left in males and 25.3±2.0 mm and 25.1±2.2 mm in females. The difference in this measurement is statistically significant (P<0.05) (Table 3).

**Table 3:** The average distances from the mental foramen to various sites. Distance from the mental foramen to the inferior border of the mandible (MF-MB) and distance from the mental foramen to the mandibular midline (MF-MM)

Distance	Gender	Distance (mm)	
		Right	Left
MF-MB	Male	15.4±2.4	15.6±2.0
	Female	14.0±2	14.3±1.6
	P value	0.031	0.016
MF-MM	Male	27.0±2.6	27.0±2.9
	Female	25.3±2.0	25.1±2.2
	P value	0.015	0.018
Independent samples test			

The results of the mean distances of the MF on both sides were calculated in relation to age groups, but no statistically significant differences were found (Table 4).

**Table 4:** The average distances from the mental foramen to various sites according to age group classification. Distance from the mental foramen to the inferior border of the mandible (MF-MB) and distance from the mental foramen to the mandibular midline (MF-MM)

	Age	MF-MB (mm)	MF-MM (mm)
Right	18-25	14.7±2.7	14.5±2.1
	26-40	14.8±2.5	15.2±1.9
	41-55	14.9±1.8	15.4±1.8
	56-69	14.5±1.9	14.8±2.0
	P value	0.989	0.447
Left	18-25	26.2±2.9	25.9±3.4
	26-40	26.1±2.5	25.9±2.6
	41-55	25.8±1.8	26.2±2.0
	56-69	28.2±1.6	29.0±0.6
	P value	0.687	0.192
ANOVA test			

## Discussion

MnF

Although IAN block is frequently used as a local anesthetic for restorative and surgical treatments of mandibular molars, it has been reported that this method is associated with the risk of clinical failures up to 15-20%, even when performed by experienced dentists. The most common cause of anesthesia technique failure is inaccurate needle placement due to the lack of precision in locating MnF anatomical structures. In addition, it could be explained that the position of the mandibular ramus and the MnF is disparate in each person. The position of the MnF affects the success of the IAN block, and changing the position of the MnF reduces the possible achievement of the procedure [9]. Therefore, the location of the MnF is critical for a successful IAN block and the prevention of complications common to maxillofacial surgery [10].

To gain a successful mandibular nerve block, it is essential to simply and reproducibly locate the MnF in CBCT, regardless of the patient's condition. The CBCT systems currently are not as large as those of traditional CT scanners along with the cost being lower. These characteristics make CBCT popular in dental clinics [9,12].

The variations in the voxel sizes of CBCT machines and the utilization of different morphological analysis software could impact the outcomes. These differences could offer an explanation for the dissimilarities observed between our research findings and those of other studies.

The average distances of the study variables concerning age groups were not found to have statistically significant differences. This could be explained by the selection of subjects requiring more than 18 years old, an age range that is not much variation in anatomical landmarks. Park et al. demonstrated mandibular growth during the maturation process [10]. The position of the MnF could be distinguished from age. In the deciduous dentition stage, the MnF is located below the occlusal plane, while in adults, it is positioned 4.14 mm higher than the occlusal plane. To minimize the potential influence of this positional change, this study only included patients aged between 18 and 31.

Ahn et al. collected CBCT data from the picture archiving and communication system of Kyung Hee University Dental Hospital, Korea, in 2020 [9]. The distance of the MnF-A was 15.3±2.7 mm in males and 15.7±2.6 mm in females. The distance of the MnF-P was 19.1±2.2 mm in males and 16.9±1.5 mm in females. It is a statistically significant difference (P<0.05). The average distance of the MnF-MN was 23.9±3.6 mm in males and 21.8±4.3 mm in females. The MnF-MI was 22.3±4.5 mm in males and 19.6±2.5 mm in females. This result shows a statistically significant difference by gender. Nevertheless, our study only showed statistically significant differences by gender in the results of the MnF-MI and MnF-MN. This is due to the differences in collection methods and demographic characteristics in each country.

Shalini et al. analyzed dried mandibular bones collected from the bone bank at Dhanalakshmi Srinivasan Medical College and Hospital, India, in 2016 and obtained the following dimensions: the MnF-A was 17.11±2.74 mm on the right and 17.41±3.05 mm on the left; the MnF-P was 10.47±2.11 mm on the right and 9.68±2.03 on the left; the MnF-MN was 21.74±2.74 mm on the right and 21.92±3.33 mm on the left; and the MnF-MI was 22.33±3.32 mm on the right and 25.35±4.5 mm on the left [20]. Dried bone samples were randomly selected, with unknown races being one of the explanations for the different results.

MF

The precise positioning of the MF is beneficial in dental science since local anesthesia of the mandible is achieved by reaching the inferior dental nerve endings placed in the MF. Therefore, in anesthesia, especially in the anesthetic area, problems occur when an accurate detection of the MF cannot be done, which could cause the administration of the anesthetic drugs in elevated dosages [4].

The findings of our study are consistent with the results of a study [16]. Their study showed that the position of the MF changes during the eruption of primary teeth and is almost stable during the eruption of primary and mixed teeth. Despite our samples being all over 18 years old, the distances from the MF to the midline and to the lower border of the mandible remain constant across ages.

According to Ahn et al., the average distance from the superior border of the MF to the lower border of the mandible (MF-MB) on the right side was 13.26 mm (SD±2.34), and that on the left side was 13.37 mm (SD±2.19). The results of the paired T-test showed no statistically significant differences in the MF-MB distance between the right and left sides. Our average results are higher than the data of this study. Furthermore, this study also found no statistically significant difference between the average age and MF-MB distance [16]. According to a study [21], the lower border of the MF lying above the lower border of the mandible was about 12.31 mm. Meanwhile, the average distance between the MF and the lower border of the mandible fluctuates about 10-15 mm in males [21]. Our results also fall within this range. These studies all show statistically significant differences between genders and the distance from the superior border of the MF to the lower border of the mandible, showing that the distance is higher in males than females.

However, the MF was located, on average, lying above the border mandible, which was around 13.2 mm. Additionally, the mean gap in 386 CBCT scans was 12.4 mm. Gaps were not significantly different at the edges, but they were significantly larger in males (13.1 mm) compared to that in females (11.8 mm) [3].

According to Pele et al., the MF is equidistant from the midline of the mandible. The average distance did not vary much between studies, about 23-26 mm [21]. According to Ahn et al. [9], the average distance from the anterior border of the MF to the midline (MF-MM) is 25.86 mm (SD±0.27) on the right and 25.53 mm (SD±0.31) on the left side. The results of the paired sample test showed no statistically significant difference between the right and left in terms of MF-MM distance (value=0.112). There is no statistically significant difference between genders in the MF-MM (right value=0.90; left value=0.58). In addition, there is no statistically significant difference between the average age and the MF-MM distance (right=−0.131, value=0.08; left=−0.122, value=0.104) [17]. Our results in both men and women are within the range of these studies. Additionally, the distance from the anterior border of the MF to the midline is not consistent with the results of a study [16], which was done on the skull. They reported that the results fell into 28 mm, 27.61 mm, and 28.52 mm, respectively. In our study, the distance was less than those reported by the above studies, suggesting a shorter distance between foramina (safe zone) in the Iranian population [16].

Limitations

This research work has encountered some difficulties since there was no previous similar research in Vietnam. During the analysis, the collected sample did not cover a wide range of ages. The data source was software with reliable parameters; thus, errors in the study are not certainly guaranteed.

## Conclusions

It could be said that CBCT provides comprehensive information about the MnF and the MF. For clinical practitioners, it is necessary to be familiar with mandibular anatomy to perform procedures such as implant placement and IAN block. This will have a great impact on the success and confidence of dentists in clinical practice.
